# Comparative Study of Endothelial Function and Uterine Artery Doppler Velocimetry between Pregnant Women with or without Preeclampsia Development

**DOI:** 10.1155/2012/909315

**Published:** 2012-07-19

**Authors:** Augusto Henriques Fulgêncio Brandão, Ludmila Maria Guimarães Pereira, Alessandra Cristina de Oliveira Gonçalves, Zilma Silveira Nogueira Reis, Henrique Vítor Leite, Antônio Carlos Vieira Cabral

**Affiliations:** ^1^Department of Obstetrics and Gynecology, Federal University of Minas Gerais, 30130-100 Belo Horizonte, MG, Brazil; ^2^Fundação de Amparo à Pesquisa de Minas Gerais (Fapemig), 30130-100 Belo Horizonte, MG, Brazil; ^3^Fetal Medicine Center Hospital das Clínicas, Federal University of Minas Gerais (UFMG), 30130-100 Belo Horizonte, MG, Brazil

## Abstract

*Background*. Poor placentation and systemic endothelial dysfunction have been identified as main events in Preeclampsia (PE). The relationship and chronology of these phenomena are important if we are to understand the pathophysiological mechanisms underlying this major clinical problem. *Objectives*. To compare the evolution of placentation and endothelial function in normotensive and preeclamptic pregnancies. *Patients and methods*. In a prospective cohort study, 59 pregnant women with a high risk of developing PE were subjected to flow-mediated dilation (FMD) and to Doppler velocimetry of uterine arteries in order to obtain their Pulsatility Index (UtA-PI). The variations in the FMD and UtA-PI values, between 16^+0^ and 19^+6^ and 24^+0^ and 27^+6^ weeks of gestation, were compared, taking PE development into consideration. *Results*. Nine patients developed PE and the other 50 women remained normotensive. At 16^+0^ to 19^+6^ weeks of pregnancy, patients that developed PE presented higher values of UtA-PI than the normotensive group, but there was no difference in FMD results between them. At 24^+0^ to 27^+6^ weeks, the patients that developed PE presented higher values of UtA-PI and lower values of FMD than the women that remained normotensive. *Conclusions*. These results corroborate the evidence that endothelial injury is secondary to poor placentation.

## 1. Introduction

Preeclampsia (PE) is a multisystemic disorder that accounts for a large number of maternal deaths in developed and developing countries worldwide [1–3]. Although its etiology remains unclear, several events in PE physiopathology are well studied and can be evaluated using biochemical or biophysical methods. In order to prevent PE complications, there are many early detection markers, which include maternal demographics, past medical, obstetric, family history, and some current pregnancy characteristics [4–6]. 

Maternal factors and history alone can be used as a PE risk stratification method. Elevated body mass index, maternal age extremes and Afro-American ethnicity are associated with a higher risk of PE [7]. Some diseases such as diabetes and chronic hypertension also significantly increase the risk [8]. The patients that present these conditions are the ones who will most benefit from a satisfactory and specific level of care, once the risk of developing PE in this groups rises threefold, reaching a PE prevalence of 45% [3]. 

Preeclampsia is essentially an endothelial disease [9, 10]. Progressive endothelial dysfunction leads to arterial hypertension, glomerular lesion, hepatic failure, and cerebral edema [11, 12]. Endothelial function can be assessed using Flow-mediated dilation (FMD) of the brachial artery, which is an ultrasonography test that evaluates endothelial response to a reactive hyperemia [13–15]. Poor placentation as a consequence of inadequate trophoblastic invasion is one of the key events of PE physiopathology, mainly in early-onset PE, and can be evaluated by the Doppler velocimetry of uterine arteries [3, 16].

A combination of maternal factors and a uterine arteries pulsatility index (UtA-PI) of 11^+0^ to 13^+6^ weeks presented good results in the prediction of early PE (onset of clinical symptoms before 34 weeks of pregnancy) [7]. The use of UtA-PI alone also did so detecting almost 95% of all cases of early PE [17]. In the second trimester of pregnancy, the uterine arteries bilateral notch combined with endothelial dysfunction were able to predict not only patients that subsequently developed PE, but also the pregnancies that were complicated by intrauterine growth restriction (IUGR) [18].

Moreover, normal pregnancy is characterized by an increase in endothelial function and a progressive reduction in the resistance of uterine vessels, due to the placentation process [19]. PE is a condition associated with poor placentation and progressive systemic endothelial dysfunction [20]. A longitudinal assessment of trophoblastic invasion and endothelial integrity might represent a way to evaluate the satisfactory evolution of pregnancy and could result in early detection of the condition that leads to the development of PE [21].

Therefore, the objective of this study is to evaluate the variations, during the course of the pregnancy, in UtA-PI and FMD in pregnant women that did not develope PE and to compare with patients that developed PE.

## 2. Patients and Methods

### 2.1. Patients

A cohort of 59 pregnant women was recruited during the first half of gestation from the high-risk prenatal care center at Hospital das Clínicas at the Federal University of Minas Gerais. Enrolment criteria were risk factors for the development of PE: chronic hypertension (9, 15.3%), pre-pregnancy mellitus diabetes (4, 6.8%), previous history of PE (12, 20.3%), family (mother or sister) history of PE (4, 6.8%), elevated prepregnancy body mass index (BMI, defined as >30 Kg/m^2^) (5, 8.5%), or primategestation (12, 20.3%). The University Ethics Committee has approved this investigation.

### 2.2. Study Design

The prospective cohort composed of the eligible pregnant women was followed from 16^+0^ to 19^+6^ weeks of gestations through to delivery. Data regarding delivery and the newborn were collected. Arterial pressure was measured twice at both arms, with at least 15 minutes between measurements and the mean arterial pressure was used in the study. The diagnosis of PE was made according to the criteria from the National High Blood Pressure Education Program Working Group in 2000—blood pressure (BP) higher than 140 × 90 mm Hg associated with proteinuria >300 mg/mL after 20 weeks of pregnancy in patients with previously normal values of arterial pressure. Superimposed PE in patients with chronic hypertension was taken into consideration when any one of the following was present: (1) severe blood pressure elevations (greater than 160/110 mm Hg); (2) heavy proteinuria (more than 2.0 grams per 24 hours); (3) blood pressure suddenly increased after a period of good control; or (4) serum creatinine increased to more than 1.2 mg/dL [22].

Dopplervelocimetry of uterine arteries and FMD were both performed twice, at enrolment moment and between 24^+0^ to 27^+6^ weeks of gestation. Pregnant women were asked to rest for at least 15 minutes before the ultrasound evaluation. A transabdominal transducer was placed on the lower quadrant of the abdomen, angled medially, and the color mode was used to identify the uterine artery at the apparent cross-over with the external iliac artery. Measurements were taken approximately 1 cm distal to the crossover point. Care was taken to ensure that the angle of insonation was less than 60°. The FMD assessment was performed using a 5 to 7 mHz linear transducer. The brachial artery in the dominant arm was identified medially in the antecubital fossa. The clearest image of the artery was scanned over a longitudinal section, approximately 5 cm above the elbow, at the end of the diastole. The measurement, performed in M-mode, was simultaneously monitored using the B-mode of the equipment to ensure that the measurement was taken during the moment that presented the lowest distension of the vessel walls, in order to avoid measurements of the larger vascular calibers originated from the systolic vascular distension. Arterial diameter was obtained from frozen screen images, by calculating the mean of three measurements of the caliber of the vessel (*D1*). After this first procedure a pneumatic cuff, placed on the forearm, distal to the ultrasound imaging site was inflated, to a suprasystolic pressure of 250 mm Hg for 5 minutes, after which, the cuff was slowly deflated. One minute after the deflation, the mean of three new measurements of the caliber of the vessel was obtained using the same technique described above (*D2*). 

Based on the previously described standards, a new measurement of the brachial artery caliber was carried out. The FMD value was obtained using the following calculation: FMD  (%) = [(*D*2 − *D*1)/*D*1] × 100, where *D*1 = basal diameter and *D*2 = postocclusion diameter.

### 2.3. Statistical Analyses

The normality of all continuous data was assessed using the Shapiro-Wilks test. For the statistical analysis, clinical characteristics, FMD, and Doppler velocimetry value variation were described and compared between groups of preeclamptic or nonpreeclamptic women using Pearson chi-square tests, for categorical variables, and the *t*-tests or Mann Whitney Test for continuous variables. Statistical significance was defined as *P* < 0.05. 

The variation in FMD between the two periods was obtained using the following formula: FMD-V (%) = [(FMD 16^+0^ to 19^+6^ − FMD 24^+0^ to 27^+6^)/FMD 24^+0^ to 27^+6^] × 100. A similar formula was used to assess the variation between UtPI results.

The statistical analysis was performed in SPSS 19 (SPSS Inc., Chicago, IL, USA).

## 3. Results

Nine of the 59 patients developed Preeclampsia: four before 34 weeks of pregnancy (early-onset PE) and 5 after 34 weeks (late-onset PE).

The demographic and pregnancy characteristics of the women are presented in Table 1, along with FMD and UtA-PI results between 16^+0^ to 19^+6^ and 24^+0^ to 27^+6^ weeks. At 16^+0^ to 19^+6^ weeks, UtA-PI was significantly higher in the group of patients that subsequently developed PE (*P* = 0.009). There was no difference between initial FMD values in the two groups (*P* = 0.350). At 24^+0^ to 27^+6^ weeks, UtA-PI was, again, higher in the PE group (*P* < 0.001). FMD values were, at this time, lower in the group that developed PE (*P* = 0.001).

**Figure 1:**
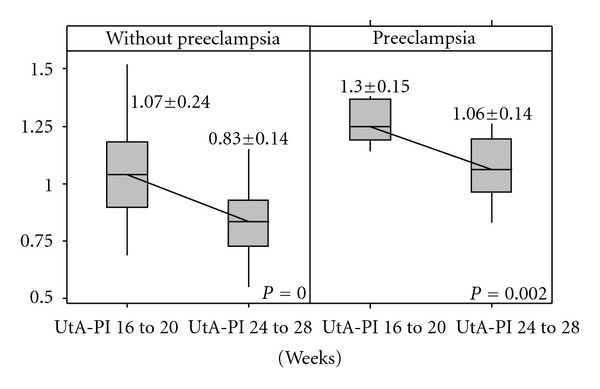
Variation of uterine arteries pulsatility index in the two groups.

When the UtA-PI results obtained in the two gestation periods of were compared, it was possible to observe that for both a nonpreeclamptic or preeclamptic evolution, values from 24^+0^ to 27^+6^ were significantly lower than the ones obtained from 16^+0^ to 19^+6^ weeks (*P* < 0.002 and *P* = 0.002, resp.—Figure 1). Additionally, there was no significant difference in the UtA-PI reduction between the non-PE group and the PE group (19.8 ± 13.3% × 17.8 ± 10.8%, *P* = 0.600).

**Figure 2:**
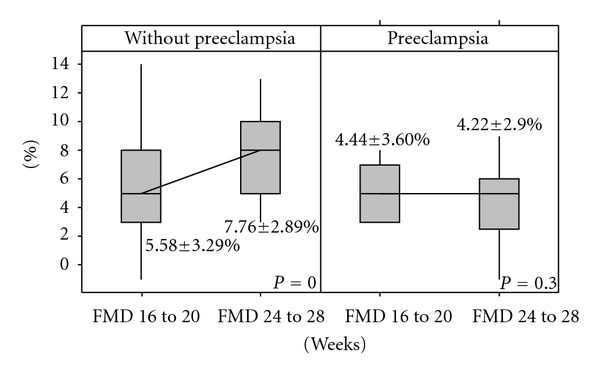
Variation of flow-mediated dilation in the two groups.

Regarding the endothelial response evaluated using FMD exams, at the second examination the rate of vasodilation after shear stress was higher in the group of patients that did not develop PE (*P* < 0.001) though this physiological phenomenon did not occur in the group that subsequently developed PE, which showed similar values when these two periods were compared (*P* = 0.300—Figure 2). The group of patients that did not develop PE presented an increase of 74.2 ± 184.2% in FMD results, while the group that developed PE presented a decrease of 16.6 ± 38.2%  (*P* = 0.003). 

## 4. Discussion

Hypertensive pregnancy disorders are an important cause of severe acute maternal and fetal morbidity and mortality. Therefore, the majority of deaths could be avoided by providing them timely and effective care [2]. In order to prevent or ameliorate PE complications, we must first understand the pathophysiological mechanisms underlying this clinical problem. This study contributes by demonstrating that, when following a high-risk pregnancy, a significant reduction in endothelial function may be detected using the brachial artery FMD test on those women that will develop PE.

The placenta is central for the development of preeclampsia. Systemic endothelial dysfunction reduces the perfusion of tissues and organs, including the placenta itself, creating an even more hypoxemic environment. Chronic placental hypoxia or alternate periods of hypoxia and re-oxygenation lead to oxidative stress, placental apoptosis and necrosis and, consequently, an increase in the expression of proinflammatory, antiangiogenic, and angiogenic factors, perpetuating systemic endothelial dysfunction. This is particularly important once it might represent a continuous cascade of pathophysiological events that only ends with the complete removal of the placenta [23]. 

The increase in endothelial function during pregnancy is an expected and well-studied event. The capacity for blood vessel dilation is a necessary condition for a healthy gestation and when absent maternal and fetal prognosis is compromised [20]. Our results showed that in patients who developed PE this phenomenon had failed, as the FMD response decreased during the second half of gestation, according to comparative results with the first half. 

On the other hand, patients, who did not subsequently develop PE presented better capacity for vessel dilation over the course of the pregnancy. It's important to point out that the results obtained from 16^+0^ to 19^+6^ were similar between the two groups (4.44 ± 3.61 versus 5.58 ± 3.29, *P* = 0.35), but a significant difference was observed from 24^+0^ to 27^+6^ weeks. With these findings we can suppose that the pathologic endothelial dysfunction in PE was clinically detectable in the second half of pregnancy, instead of the first. This statement is particularly important since it might indicate that poor placentation during the first trimester of pregnancy in PE pregnant women occurred before systemic endothelial dysfunction, which was probably secondary to the structural placental vessels abnormalities. 

Regarding the perfusion of uterine arteries we identified higher UtA-PI values in the group that developed PE, from 16^+0^ to 19^+6^ weeks on. These results were similar to previous reports by Llurba et al. [16] and Onwudiwe et al. [7]. On the other hand, there were no significant differences between the variations over the course of the pregnancy in UtA-PI in the normotensive or preeclamptic groups. The results could lead to an interpretation of poor placentation as an early condition in pregnancies complicated by PE. 

The present study was carried out to follow a population at high risk of developing PE. Some pregnant women presented conditions associated with endothelial dysfunction prior to pregnancy such as chronic hypertension and Mellitus Diabetes and some had risk factors not related to endothelial injury or dysfunction, such as prior or family history of PE. From one point of view, we created a heterogeneous group, but with a similar risk of PE development, this selection criteria reflects the real obstetric demand for the prenatal care. This fact could explain the reason why FMD results in the group without subsequent development of PE are lower than other reports that used the same indirect endothelial evaluation as reported by Takase et al. [24]. Actually, during 24^+0^ to 27^+6^ weeks of gestation, the PE condition could reduce endothelial function even more, as demonstrated by a lack of variation in vasodilation after shear stress. Future studies could evaluate if there are differences in the variation in FMD values between groups of patients with a presence or absence of prepregnancy diseases that subsequently developed PE.

In this cohort, five out of nine pregnant women developed late-onset PE and had an inappropriate endothelial function increase, evaluated using FMD. A new theory points to an intrinsic failure in trophoblast differentiation at different time points. This may lead to either a mild disorder with late-onset appearance, or intrauterine growth retardation, with or without maternal symptoms. Early-onset PE might be a result of an affected Villous Cytotrophoblast or Syncytiotrophoblast differentiation, while the late onset form is a consequence of extrinsic factors, which create an overload in placental apoptotic fragments. Once necrosis is the final pathway of these two processes and a cause of an inflammatory response associated with endothelial dysfunction, this theory could explain why patients with early or late forms of PE presented lower values of FMD [23]. 

The assessment of endothelial function through FMD has proved to be a reliable method of identifying endothelium integrity [15, 25]. In this study, FMD was only used to conduct an indirect evaluation of endothelial function, but as a next step, biochemical markers such asymmetric dimethylarginine (ADMA) [18], angiogenesis factors and vasoactive peptides might be additionally used for this purpose and could be used to validate our findings. Also, we strongly suggest that a new FMD evaluation should be performed at more advanced gestational ages in new studies. This would be particularly important in the evaluation of patients that subsequently develop the late-onset form of PE. 

In conclusion, our results demonstrated that early identification of compromised UtA-PI was related to bad prognosis of systemic arterial pressure during pregnancy and clinically corroborated the importance of poor placentation in PE. A lack of variation in FMD during pregnancy was observed in patients that developed PE, in contrast with the increased physiologic endothelial function in normotensive pregnancies. This clinical point of view of the endothelial function placed the endothelial injury as a secondary event to an inadequate trophoblast invasion. As the vascular endothelium plays an active role in the control of homeostasis and thrombosis and influences vascular tone [9, 25], FMD evaluation could be a potential PE marker for pregnancies presenting a high risk for this condition. 

## Figures and Tables

**Table 1:** Clinical characteristics and ultrasound parameters of pregnant women cohort, according to preeclampsia development.

	Pregnant women without PE (*n* = 50)	Preeclamptic women (*n* = 9)	*P *value
Maternal age (years)	29,4 ± 6,5	29,4 ± 5,0	0,99^∗∗^
Body mass index (kg/m^2^)	25,0 ± 6,6	27,5 ± 7,6	0,32^∗∗^
Obese pregnant women	7 (14%)	2 (22%)	0,24^∗∗∗^
Number of gestations	2 (1–8)	2 (1–5)	0,27^∗∗^
Primiparous	16 (32.0%)	3 (33.3%)	0,17^∗∗∗^
Ethnics: caucasian	15 (30%)	2 (22%)	0,42^∗∗∗^
Ethnics: Afro-American	13 (26%)	2 (22%)	
Ethnics: other	22 (44%)	5 (56%)	
Gestational age at enrolment (weeks)	17,6 ± 1,4	17,0 ± 1,3	0,24^∗^
Mean arterial pressure at enrolment (mm Hg)	90,6 ± 7,8	92,2 ± 6,0	0,55^∗^
Gestational age at second examination (weeks)	25,8 ± 1,2	25,6 ± 0,9	0,60^∗^
Mean arterial pressure at second evaluation (mm Hg)	83,8 ± 7,34	88,1 ± 9,0	0,12^∗^
Gestational age at delivery (weeks)	39,4 ± 0,9	35,0 ± 1,7	0,00^∗^
UtA-PI at enrolment	1,07 ± 0,24	1,29 ± 0,15	0,01^∗^
UtA-PI at second evaluation	0,83 ± 0,14	1,06 ± 0,14	<0,001^∗^
UtA bilateral diastolic notch at second evaluation	8 (16%)	7 (78%)	<0,001^∗^
Basal diameter of brachial artery at enrolment (mm)	3,32 ± 0,47	3,34 ± 0,54	0,82^∗^
Flow-mediated dilation between at enrolment (%)	5,58 ± 3,29	4,44 ± 3,61	0,35^∗^
Basal diameter of brachial artery at second evaluation (mm)	3,36 ± 0,42	3,40 ± 0,49	0,81^∗^
Flow-mediated dilation at second evaluation (%)	7,76 ± 2,89	4,22 ± 2,90	0,001^∗^

Note: gestational age of enrolment: 16^+0^ to 19^+6^ weeks. Gestational age of second evaluation: 24^+0^ to 27^+6^ weeks . PE: preeclampsia, ^∗^Student's *t*-test, ^∗∗^Mann-Wthitney *U* test, ^∗∗∗^Chi-square test.
